# Experts and Novices Use the Same Factors–But Differently–To Evaluate Pearl Quality

**DOI:** 10.1371/journal.pone.0086400

**Published:** 2014-01-22

**Authors:** Yusuke Tani, Takehiro Nagai, Kowa Koida, Michiteru Kitazaki, Shigeki Nakauchi

**Affiliations:** 1 Department of Computer Science and Engineering, Toyohashi University of Technology, Toyohashi, Aichi, Japan; 2 Graduate school of Science and Engineering, Yamagata University, Yonezawa, Yamagata, Japan; 3 Electronics Inspired-Interdisciplinary Research Institute, Toyohashi University of Technology, Toyohashi, Aichi, Japan; National Institute of Mental Health, United States of America

## Abstract

Well-trained experts in pearl grading have been thought to evaluate pearls according to their glossiness, interference color, and shape. However, the characteristics of their evaluations are not fully understood. Using pearl grading experiments, we investigate the consistency of novice (i.e., without knowledge of pearl grading) and expert participants’ pearl grading skill and then compare the novices’ grading with that of experts; furthermore, we discuss the relationship between grading, interference color, and glossiness. We found that novices’ grading was significantly less concordant with experts average grading than was experts’ grading; more than half of novices graded pearls the opposite of how experts graded those same pearls. However, while experts graded pearls more consistently than novices did, novices’ consistency was relatively high. We also found differences between the groups in regression analyses that used interference color and glossiness as explanatory variables and were conducted for each trial. Although the regression coefficient was significant in 60% of novices’ trials, there were fewer significant trials for the experts (20%). This indicates that novices can also make use of these two factors, but that their usage is simpler than that of the experts. These results suggest that experts and novices share some values about pearls but that the evaluation method is elaborated for experts.

## Introduction

Pearls are known as jewels from the bottom of the sea. Their mystique from being produced by shellfish and their lustrous iridescence has attracted many people worldwide. The pearls produced by Akoya pearl oysters (*Pinctada fucata martensii*) have superior luster and impressive iridescence. In addition to these two features, their size, roundness, and the existence of scars or pocks are the key features inspected by farmers, traders, and craftsmen, who are collectively addressed as “experts” [1]–[3]. The quality or value of pearls is decided only by well-trained experts’ visual inspection at north-facing windows on sunny mornings or afternoons. Further, consumers and novices accept these decisions. This situation suggests interesting questions: How do experts use visual information to evaluate pearls? What do they learn? What supports this tacit agreement between experts and novices?

About 100 years ago, pearl farming–a practice whereby Akoya pearl oysters are cultured and the spherical pearls are constantly harvested from them–began in Toba, Mie Prefecture, Japan. Even now, Toba is one of the principal areas of Akoya pearl farming and manufacturing.

**Figure 1:**
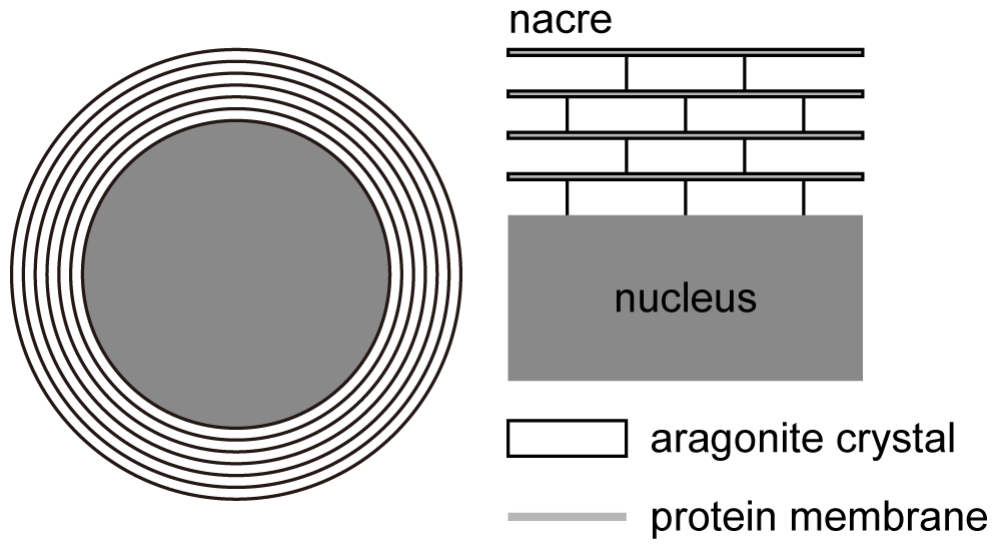
Schematic diagram of internal structure of cultured pearl. The translucent nacre surrounds the nucleus. The nacre comprises hundreds to thousands of nacreous layers. The thickness of each layer is approximately 300–500 nm.

A cultured pearl consists of a nucleus surrounded by hundreds to thousands of translucent layers of nacre. The nucleus is a spherical bead made of shell, and the nacre is a secretion of pearl oysters consisting of calcium carbonate (CaCO_3_) and proteins like conchiolin. Calcium carbonate is an ingredient of both the nucleus and nacre; the former is calcite crystal, whereas the latter is aragonite crystal. The thicknesses of the aragonite crystal and the protein membrane are approximately 300–500 nm and 10 nm, respectively. Thus, the thickness of a nacreous layer is in the range of the wavelength of visible light (Figure 1). These characteristics of the nacre are the origin of pearl’s iridescence, one of the essences of pearliness. That is, the lustrous iridescence of pearl is due to the interference color, which is a kind of structural color caused by the multilayer thin film structure.

The strength and chromaticity of pearls’ interference color depend on the thickness of the nacre layers and the length difference between the optical paths, respectively. Incident light travels through nacre in a complex way because of multiple reflections, refractions, and penetration in each nacreous layer. Therefore, the interference color is independent of the direction of the light source, and it depends on the viewing direction and the thickness of each nacreous layer [2], [3]. As a result, nearly concentric chromatic patterns are seen on spherical pearls. In general, pearls regarded as good by experts have a typical concentric chromatic pattern, changing from greenish in the center to pinkish at the periphery [3].

Both the chromatic pattern and chromaticity of pearls’ interference color correlate with the physical structure of the nacre; thus, experts evaluate the physical regularity of the pearls, in a sense. This leads to the following questions about novices, however: Can they evaluate pearls the same way as the experts do? If so, what differentiates the experts from novices?

In general, experts’ senses seem superior to those of novices. For instance, most people believe that only experts can detect certain qualitative differences. Furthermore, judgments made by well-trained experts are often expected to be identical.

However, according to research on differences between experts and novices, such as that on athletes and artists, experts do not exceed novices in terms of lower-level abilities [4]–[6]. For example, the perception of both expert and novice painters was distorted by size constancy in a similar way [6]. The superiority of experts appears to manifest in a limited number of cases or situations [7]. That is, most distinctions can probably be attributed to differences in strategy or cognitive level [8]. In addition, top-level athletes have been found to have special learning abilities [9].

Whether concordance among experts is observed depends on the domain. For example, sommeliers and wine tasters outperform novices in olfactory discrimination and matching [10]. Well-trained tasters are able to rate the concentration of sodium chloride in mixtures of sodium chloride and sucrose solutions more correctly and consistently than novices can [11]. On the other hand, experienced violinists are divided in terms of preference for the tonal quality of violins [12].

In this paper, we compare experts and novices in terms of preference and within-individual consistency of pearl evaluation. If the tendency of rank ordering by novices resembles that of experts, then the experts’ evaluation rules could be attributed to an innate sense of beauty, which should be shared by experts and novices; if not, then novices’ rank-ordering tendency is likely artificial. Furthermore, if the consistency of rank ordering by the novices approaches chance levels, then the criterion they use is vague or unconscious; if it is high, the criterion that they use should be obvious. If the experts’ consistency is higher than that of novices, experts indeed have elaborated the pearl evaluation method. Further, we investigate the functional relationship between pearl evaluation and optically measured glossiness and interference color that previous researchers [1]–[3] and experts have reported to be the factors considered in the evaluation of a pearl’s quality.

## Materials and Methods

### Participants

Eight experts (mean = 43.63, SD = 4.56 years old) and eleven novices (mean = 41.73, SD = 4.96 years old) participated in the experiment. All of them were males and had normal or corrected-to-normal vision. The expert participants included five pearl oyster farmers with more than 20 years of experience in the field; two pearl marketers with more than 15 years of experience; and one scientist who had been studying improvements in the health of cultured pearls in order to raise the quality of the pearls generated in pearl oyster farming for five years. All experts worked in Toba, Mie Prefecture, Japan. Although there are no national qualifications or licenses concerning pearls in Japan, in this study, experts are considered to be individuals who had learned about pearls from masters of the field, had been handling pearls in their work as professionals for a long period, and who were recognized as full-fledged experts by other experts.

The novice participants included ten scientists affiliated with Toyohashi University of Technology as assistant professors, associate professors, and professors and one university clerk. All of them were unfamiliar with pearls in both their research activities and daily life and were unaware of the purpose of the experiment. After the experimental procedure was explained, all participants gave written informed consent before the experiment began. This study was approved by the Committee for Human-Subject Studies at Toyohashi University of Technology.

### Stimuli

We used 20 Akoya cultured pearls that had been labeled A-rank and B-rank. We got them from the trader in Kobe, a major center of pearl circulation (These pearls we used were labeled by some experts in Kobe other than our expert participants). All pearls used in the experiment were approximately 8 mm in diameter. The pearls were arbitrarily placed into two sets, with each set consisting of five A-rank (“good”) pearls and five B-rank (“fair”) pearls. Although Toyota and Nakauchi [3] used pearls ranging between A-rank to C-rank (“bad”), we avoided C-rank pearls because a preliminary examination revealed that C-rank pearls were easily distinguished from A- and B-rank pearls. To identify each pearl, a small (1 cm × 1 cm) piece of white paper, on the back of which an identification code was written, was attached to each pearl. The interference color and glossiness of each pearl were quantified using a device developed in our laboratory [3], which simultaneously provides both quantified interference color and glossiness.

**Figure 2:**
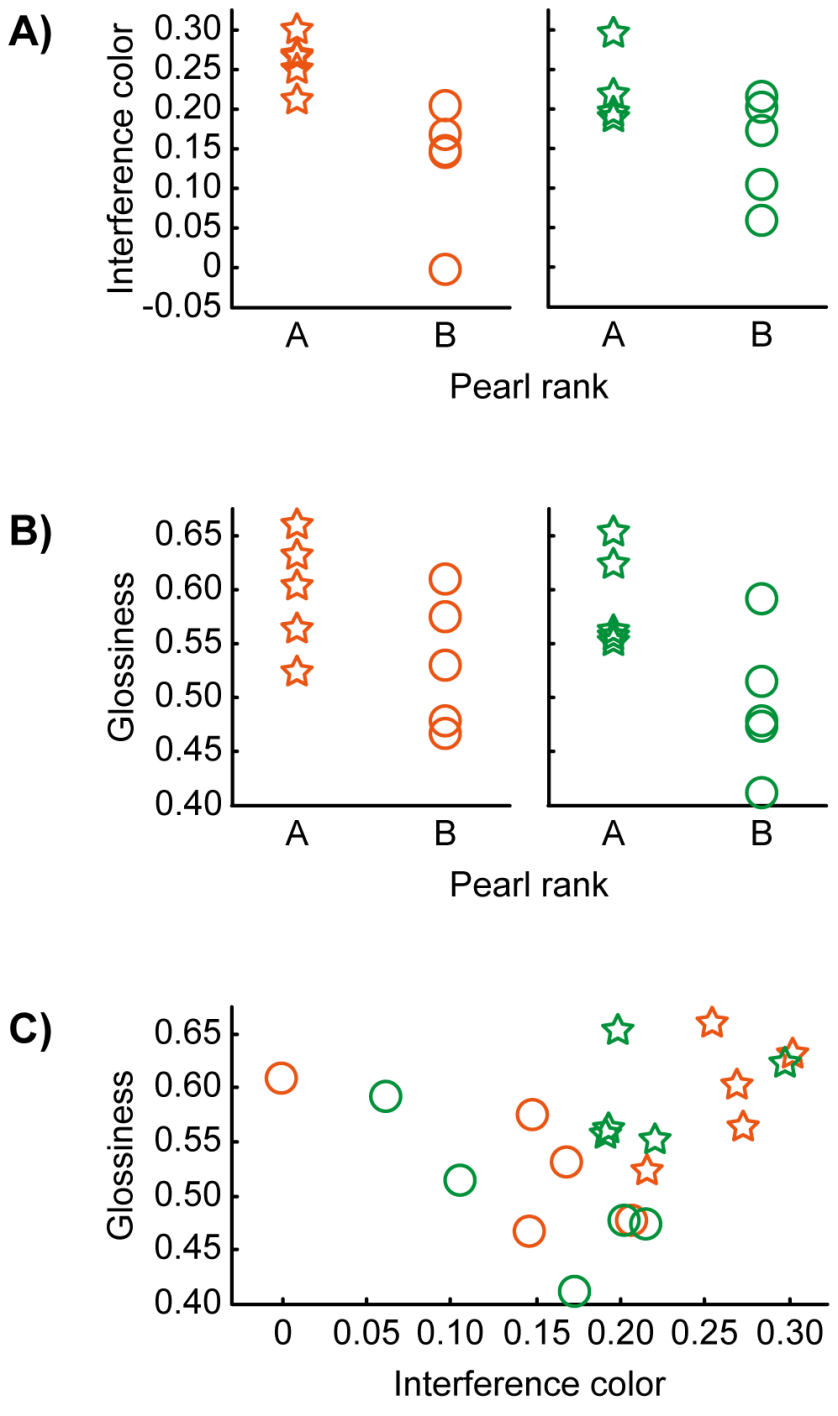
Measured values of interference color and glossiness. Each point shows the average values of 15 measures of a pearl. We used five pearls for each rank (“A” rank shown by star and “B” rank shown by circle) in both sets. Higher measured values seem to be associated with higher ranks (“A” rank) in both interference color and glossiness. (A) Relationship between interference color and pearl ranks in each stimulus set. (B) Relationship between glossiness and pearl ranks in each stimulus set. (C) Relationship between interference color and glossiness. Colors represent stimulus set. Pearson’s *r* of the set in orange is 0.208, and that shown in green is 0.128.

In the device for qualifying pearls, a pearl is illuminated from the opposite side of the surface from where the camera is placed, and a transmission image is captured. The intensity map of the transmission image is calculated using both white and very narrow band light (around 520 nm). The device then calculates the intensity gradient with eccentricity using weighted coefficients; the resulting value is a quantitative measurement of interference color [3]. The skewness of the luminance histogram is used as the quantification value for the glossiness. Although Anderson et al. have emphasized that skewness is not a cue for perceptual glossiness [13]–[17], the correlation between skewness and glossiness–first discovered by Motoyoshi et al. [18]–was confirmed by the inventors using pearls ranging from A-rank to C-rank. The pearl was illuminated from the same side of the surface from where the camera is placed, and the image is captured. The skewness of the luminance histogram of the region where the pearl is in the image is calculated. We measured interference color and glossiness from fifteen points of view or directions and averaged them for each pearl (Figure 2).

### Apparatus

The illumination we used had the same spectral pattern as sunny afternoon light, ranging from 370 nm to 780 nm (SERIC Ltd. SOLAX XC-100AF). We intended to imitate the lighting conditions under which experts look at pearls. We used two lamps and a diffuser to illuminate the desk; the distance between the lamps and the desktop was 100 cm. The illuminance on the desk was 109 lx.

**Figure 3:**
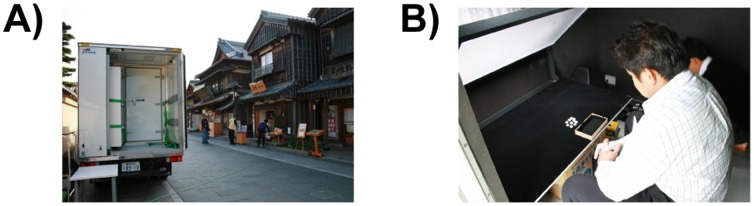
Photographs of the experimental conditions. (A) A snapshot of “Mobile-Labo” truck. The experimental space is beyond the inner door. For this photograph, we parked on a street in front of the office in the courtyard of the laboratory and conducted the experiment. (B) Experimental setting for the experts. The person sitting in front is the participant. The white board above the black desktop is the diffuser. The participant is asked to sort the pearls according to a subjective criterion in the small box. The experimenter received the box and recorded the order.

The experiment was conducted in the cargo space of a truck that had been modified for use in psychological experiments, called Mobile-Labo (Figure 3). Mobile-Labo enabled us to quickly perform the experiments, which prevented the pearls from degradation through aging. Because it was harvesting season for pearl farming and the experts were extremely busy, we visited their farms, offices, and institutes individually in this truck. The novice participants also performed the experiment in Mobile-Labo.

### Procedure

The task was to evaluate ten pearls, which were randomly placed on a black desk mat, according to their goodness. The novices were not given any instruction about how experts grade pearls; they evaluated pearls according to their own subjective criteria or preference. On the other hand, the experts were asked to evaluate pearls according to their professional criteria. Although the experts group included participants with different profession and this could cause the diversity of criteria in this group, the economical relationships between their works were very close. For example, marketers selected and bought pearls selected by farmers. If there were great differences between their evaluations, the commercial transactions would never be established. That is, we regarded the difference in their criteria derived from the difference in their profession as allowable. Although roundness and the existence of scars or pocks also affect the goodness of pearls, the participants were asked to ignore these aspects. They were allowed to look at the pearls while changing viewing positions or moving their heads, but were not allowed to pick up the pearls.

The participants observed and compared ten pearls, and then sorted the pearls by subjective rank order of goodness. After the participant declared that the ordering was complete, the experimenter (who sat next to the participant) checked the identification codes and recorded the rank order. The participants repeated this task five times for each set, alternating between sets. In total, the participants repeated this task ten times. They were not given any feedback about their ordering during the experiment.

## Results

**Figure 4:**
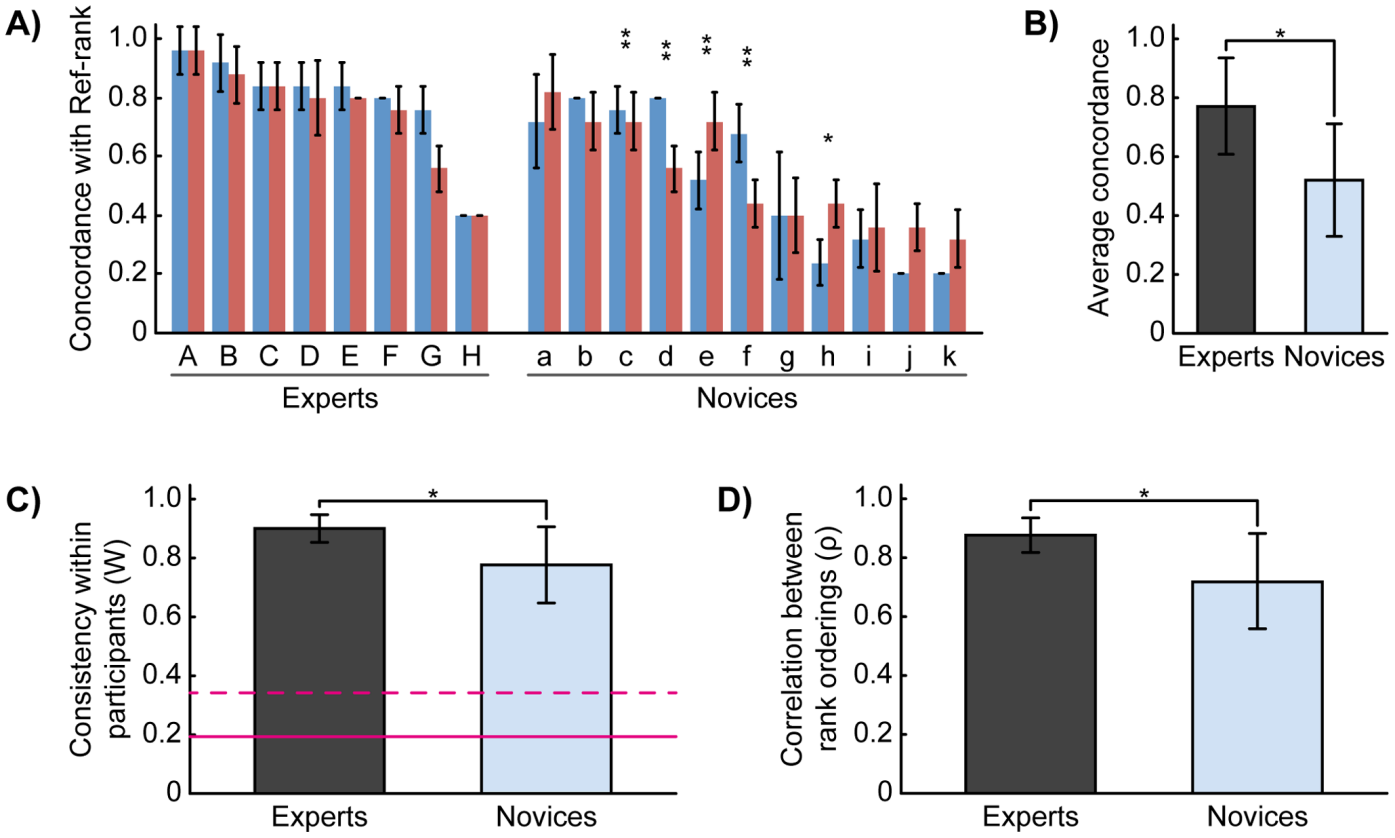
Comparisons between experts and novices. (A, B) Individual averages of concordance rates for each pearl set (blue and red). In A, the participants are sorted into high to low concordance. The asterisks signify that the difference between concordances for each pearl set was significant (*: *p*<0.05; **: *p*<0.01). (C) The average concordances of both groups are shown. As shown, there is a significant difference between them. (D) For consistency, the values of experts and novices were significantly higher than chance and the 95% upper limit. The red solid and dashed lines indicate the expected chance value of *W* and the values corresponding to the 95% upper limit of chance value of *W*, respectively, which was calculated from a randomized resampling (n = 1,000,000). The difference between them is statistically significant (*: *p*<0.05). (E) The averages of all ρ coefficients in both groups. The difference between them is statistically significant (*: *p*<0.05). The error bars in graphs refer to the standard deviations.

To compare the results between novices and experts, we first calculated the expert participants’ average ranks for each pearl. Hereafter, these averaged ranks are referred to as the “reference rank” (or Ref-rank in Figure 4A). If a pearl ranked in the first half of the reference rank was ranked from first to fifth in a trial, or if a pearl ranked in the last half of the reference rank was ranked between sixth and tenth in a trial, the ordering was regarded as concordant with the reference rank. That is, we judged the concordance of each participant’s rank ordering as whether it was categorically concordant with the reference rank; this was because experts’ daily work is to categorize pearls according to their quality. Thus, we assessed the overall concordance of each trial by determining the rate of concordant orderings. Figure 4A shows individual experts’ and novices’ average concordance for each pearl set. Most experts, except one, showed high concordance; in contrast, around half of the novices (5 out of the 11) showed concordance rates of less than 0.5. According to two-way repeated analyses of variance (ANOVAs), there were statistical differences in experts’ average concordances between pearl sets (M = 0.795 and 0.750, SD = 0.161 and 0.171, respectively; *F*(1, 32) = 5.786, *p* = 0.022); however, novices’ concordances did not differ between pearl sets (M = 0.513 and 0.533, SD = 0.237 and 0.173, respectively; *F*(1, 44) = 0.725, *p* = 0.399). Although the differences among individuals were significant in both groups, the interaction was significant only in the novices (*F*(1,10) = 4.933, *p*<0.001). That is, all experts evaluated both sets roughly equal from the point of view of concordance, on the other hand, the evaluations of some novices differed between pearl sets (shown by asterisks in Figure 4A). The group averages approached 0.773 (SD = 0.163) for experts and 0.522 (SD = 0.192) for novices, and there was a statistical difference between these averages (Figure 4B; an independent two-sample *t*-test, *t*(17) = 2.832, *p* = 0.012, *d* = 1.39).

Next, we compared the consistency of rank orderings between these two groups. Consistency referred to the similarity in rank orderings between sets of pearls within each participant. First, we calculated Kendall’s coefficient of concordance (*W*) as an index of participants’ consistency across five repeated rankings for each pearl set. Thus, we calculated two *W* values for each participant. Participants’ within-subjects consistency was thus the average of the *W*s (Figure 4C). These averaged consistencies in both groups were significantly higher than the chance *W* (red solid lines in Figure 4C). The significance was assessed by comparison between observed *W* and the 95% upper limit of a chance *W* (red dashed lines in Figure 4C). Although novices’ average consistency was relatively high (M = 0.778, SD = 0.130), it was still significantly lower than that of experts (M = 0.903, SD = 0.048; an independent two-sample *t*-test, *t*(17) = 2.449, *p* = 0.025, *d* = 1.22). Secondly, we calculated Spearman’s rank correlation coefficients (*ρ*) between the pairs of trials for each pearl set. That is, 20 separate *ρ* coefficients were calculated for each participant. The averages of the *ρ* coefficients are shown in Figure 4D. A Mann-Whitney *U* test again revealed that the experts’ consistency was statistically higher than that of novices (M_experts_ = 0.878, SD = 0.060, M_novice_ = 0.721, SD = 0.163, respectively; *U*(8, 11) = 19, *p*<0.05). The significance was confirmed after a z-transformed comparison of the correlation coefficients (an independent two-sample *t*-test; *t*(17) = 2.519, *p* = 0.045, *d* = 1.24).

**Figure 5:**
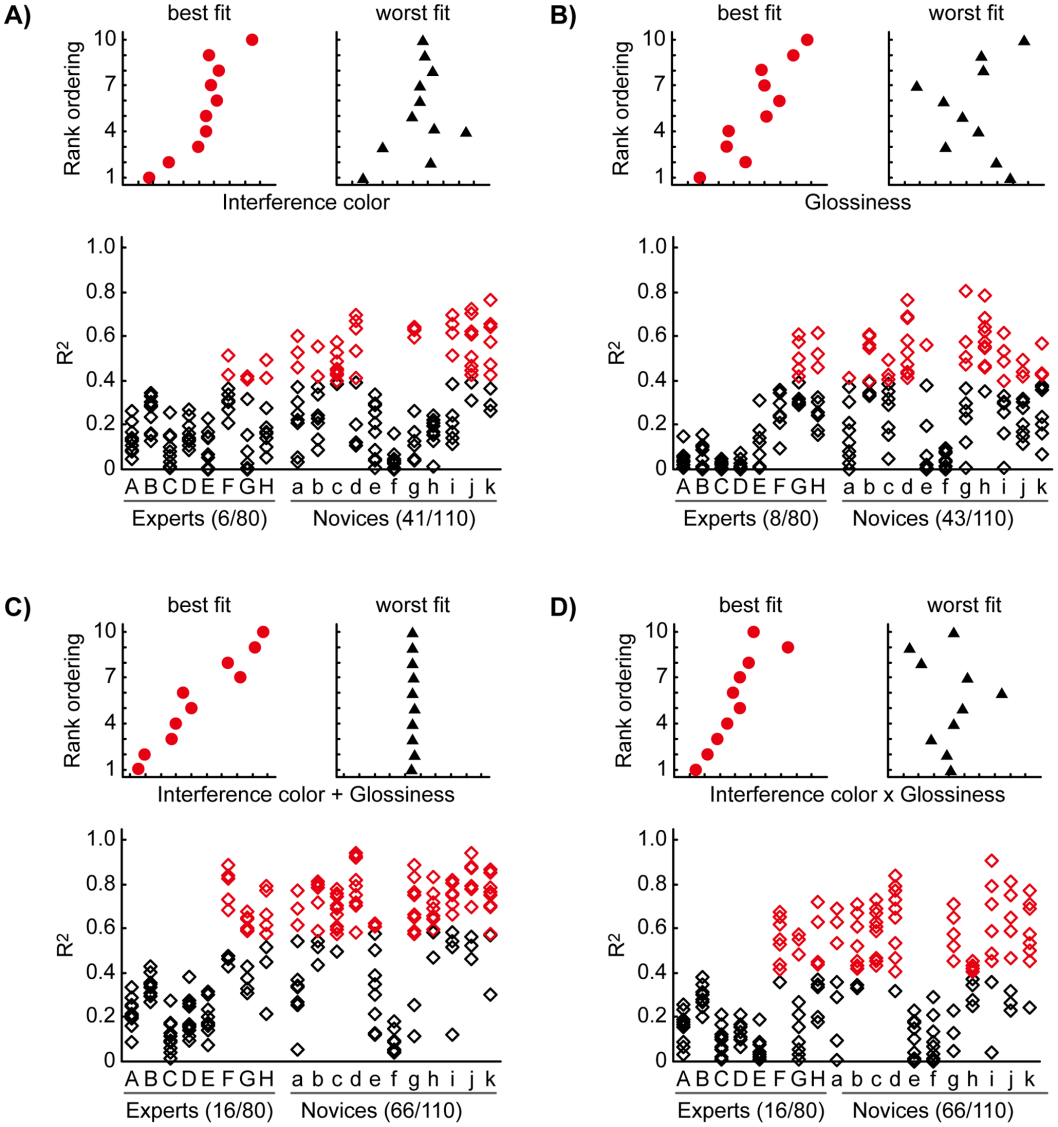
Regression analyses by measured variables. In all panels, the upper insets show the best (left) and worst (right) regression results. The vertical axes show the rank orderings, and the horizontal axes show either the explanatory variable (in A, B, D) or the prediction from the regression equation (in C). The lower graphs show the results of all the regressions. In all graphs, each point represents one trial, and each row represents one participant. The numbers written in parentheses are the numbers of trials for which the regression is significant (shown by red points) and the total number of trials. The vertical axes show the coefficients of determination (R^2^). (A and B) Results of simple linear regression analyses on interference color and glossiness, respectively. (C) Results of multiple regression analyses on both variables separately. (D) Results of simple linear regression analyses on the product of both variables.

Finally, comparisons were made between subjective grading and pearls’ optical properties. To examine whether the rank orderings were explained by the physical properties of the pearls, we carried out four regression analyses for each trial; three simple linear regressions and one multiple regression, in which optically measured interference color and glossiness were used as explanatory variables. Although a single multiple regression with two variables would have sufficed, we wanted to demonstrate to what extent each parameter (interference color, glossiness, and the product of these two variables) could explain each trial. Thus, we conducted three simple regressions as well. First, we performed separate simple linear regressions with interference color (Figure 5A) and glossiness as explanatory variables (Figure 5B). Both simple regressions showed that the rank orderings could not be fully explained by a single explanatory variable. Only 6 of 80 (7.5%) trials for the experts and 41 of the 110 (37.3%) trials for the novices showed significant correlations between the rank ordering and the interference color (Figure 5A), and 8 of 80 (10.0%) trials for the experts and 43 of 160 (39.1%) trials for the novices showed significant correlations between the rank ordering and the glossiness (Figure 5B). Next, we carried out a multiple regression analysis for each trial. That is, we tested the hypothesis that a pearl’s rank was determined by a linear combination of its interference color and glossiness. We found that the regression was significant in only 20.0% (16 of 80) and 60.0% (66 of 110) of trials among experts and novices, respectively (Figure 5C). Finally, we conducted a simple linear regression analysis in which the product of the values of interference color and glossiness was used as an explanatory variable, following the notion that interference color and glossiness cannot be segregated perceptually. The regression was significant for 20.0% (16 of 80) and 60.0% (66 of 110) of trials among experts and novices, respectively (Figure 5D). The trials in which the regression analyses were significant were found in the experts who showed relatively low concordance and in all novices with the exception of the participant “f” who showed moderate concordance. Then, we calculated the correlation coefficient between participants’ concordance level and the number of trials where the regression was significant. For the experts, the correlations were not found in any regressions. On the other hand, for the novices, positive correlations (0.44 ∼ 0.54) were found in all regressions, however, all correlations were not statistically significant (*p* = 0.08 ∼ 0.18).

## Discussion

In this study, we found that the average rank ordering of novices was significantly less concordant with the standard ranks than that of experts. Indeed, more than half of novices showed a concordance rate lower than 0.5 (i.e., only 50% of rankings were concordant). Thus, their rank orderings were apparently the opposite of the standard ranks for experts in the field, suggesting that the experts’ method of evaluating pearls or pearliness is not used by novices who had not been given any instructions about how to evaluate pearls.

Thus, novices could be divided into two groups; those whose rank orderings showed the same tendency as the standard ranks, and those whose rank orderings showed the opposite tendency. In other words, novices may use the same criteria as experts for evaluating pearls, but they utilize these criteria differently.

Interestingly, experts’ average concordance was not very high. This suggests that experts’ evaluations were not identical. One possible reason for this was that they were unaccustomed to experimental settings such as ours. Although our experimental procedure and environment were designed to imitate the conditions in which experts usually engaged in their daily work, some residual differences might have affected their performance. For example, their work task may be to categorize large numbers of pearls in A-rank, B-rank, or C-rank, while in our experimental task, experts had to rank individual pearls. Thus, the difference between categorization and rank ordering could have been larger than expected. The other candidate should be the variety or complexity of the pearl evaluation. We took five factors into consideration; size, roundness or shape, the existence of scars or pocks, interference color, and glossiness. We controlled the former three factors in choosing pearls and instructed participants to ignore them, and we tried to explain the rank orderings by optically measured interference color, and glossiness (the latter two variables). However, experts might have examined other factors that we had not considered. For example, one expert had a rank-ordering tendency that was opposite that of the other experts. In addition to the factors we had considered, he might have strongly depended on other factors that we did not consider, which would have caused his results to differ from the reference rank.

We should note that there seems to be no absolute scale of pearl beauty, as is the case for music [12]; this can be attributed to learning methods, especially reward or positive feedback. The effects of reward and positive feedback would be broad, not restricted [19], [20]. If the conditions in which the reward is given can be defined strictly, like in discrimination [10], [11], then the effects of reward would become concentrated, and some specific responses would be enhanced. On the other hand, if the condition in which the reward is given cannot be defined strictly, as in judgments of goodness or beauty [12], then the effects of reward would be diffuse. The evaluation of pearl and our results would correspond to the latter case.

Both experts and novices showed sufficient consistency in their rank orderings of pearls. However, their consistencies were not equal; the experts’ consistency was significantly higher than that of the novices. In addition, during introspection after the experiment, most of the experts noticed that only two sets of pearls were presented repeatedly. On the other hand, none of the novices said that they noticed this. This indicates that the experts were superior in terms of consistency. This could also indicate a better recognition of the pearls by experts. This could be explained by a better attention to specific pearls’ physical features, that novices did not notice, and that experts would have used in their judgment.

A series of regression analyses did not fully support the previous findings that experts use both interference color and glossiness in their judgments [1]–[3]. However, usage of these two qualities may occur in a complex, nonlinear way, because experts’ rank orderings could not be fully explained by a linear combination of the two variables. As such, experts likely employ other variables or nonlinear processing for which we did not account. In the context of perceptual learning, Watanabe et al. [21], [22] revealed that conscious effort is not essential in processing counterintuitive facts and that implicit processing has a more significant role in perceptual learning. Experts are likely to have received intensive training in their field, with or without conscious effort. Therefore, there could be other variables that the experts themselves are not aware of. On the other hand, more trials of the novices could be explained by two variables in a linear fashion. That is, the novices might evaluate pearls in a simpler manner than the experts do.

In this paper, we investigated how experts and novices evaluate pearls, although this problem had already been investigated in different ways [1], [2]. Using a semantic differential method and a multivariate analysis, Nagata et al. extracted the deciding factors used by experts in judging pearls [1]. They also examined pearl-like quality as assessed by novices in a paired comparison experiment [2]. Their aim was to model, visualize, and synthesize computer graphics of pearls; thus, their work focused on the visual features that distinguish pearls from other materials. On the other hand, this paper focused on the visual features that decide the goodness of pearls. We introduced optically measured and quantified variables to explain pearl grading. A new technique [3] enabled us to do this, and this paper is the first attempt to explain pearl evaluation quantitatively. Our data suggested that the experts’ conception of pearl goodness was somewhat accepted by the naive participants (at least half of them), who were the same as most consumers. This means that consumers accept expert-decided pearl values not only for economic reasons but also because of individual aesthetic preferences. Our data also suggested that naive participants use pearls’ interference color and glossiness to evaluate them, but probably in a simpler way than experts do. However, we do not yet have an explicit explanation for the experts’ usage of interference color and glossiness. This is the target of our future work.
